# Anatomical Variations in Critical Structures in Esophageal Surgery: Implications for Personalized Surgery

**DOI:** 10.3390/jpm16060291

**Published:** 2026-05-27

**Authors:** George Triantafyllou, Adam Mylonakis, Nikoletta Dimitriou, Chrysovalantis Vergadis, Orestis Lyros, George Tsakotos, Maria Piagkou, Dimitrios Schizas

**Affiliations:** 1 Department of Anatomy, School of Medicine, Faculty of Health Sciences, National and Kapodistrian University of Athens, 11527 Athens, Greece; georgerose406@gmail.com (G.T.); gtsakotos@gmail.com (G.T.); mapian@med.uoa.gr (M.P.); 2 First Department of Surgery, National and Kapodistrian University of Athens, Laikon General Hospital, 11527 Athens, Greece; adam.mylonakis@gmail.com (A.M.); nicole-demetriou@outlook.com (N.D.); 3 Third Department of Radiology, National and Kapodistrian University of Athens, Laikon General Hospital, 11527 Athens, Greece; valvergadis@gmail.com; 4 Fourth Department of Surgery, National and Kapodistrian University of Athens, Attikon University Hospital, 12462 Athens, Greece; lyrosorestis@gmail.com

**Keywords:** anatomical variation, esophageal surgery, thoracic surgery, aberrant subclavian artery, recurrent laryngeal nerve, thoracic duct, precision surgery, patient-tailored planning, surgical anatomy

## Abstract

Esophageal cancer remains a global challenge, with poor overall survival despite advances in multimodal therapy. Surgical resection continues to be the main curative treatment, yet esophagectomy is among the most technically challenging oncological procedures due to the esophagus’s location within the densely packed mediastinal corridor. Critical vascular, neural, and lymphatic structures surround the esophagus, and their frequent anatomical variations pose significant risks during mobilization, lymphadenectomy, and reconstruction. This review synthesizes current evidence on the anatomical variability in the vessels, nerves, lymphatics, and fascial compartments relevant to esophageal surgery. Particular emphasis is placed on aberrant arterial and venous patterns, recurrent and non-recurrent laryngeal nerve pathways, thoracic duct variants and atypical courses, and the fascial planes that are used to define surgical boundaries. By shifting the surgical paradigm from standardized anatomical assumptions to patient-specific structural mapping, we highlight how understanding these variations is driving the field of personalized surgical medicine. By integrating these anatomical insights with surgical approaches—including right and left transthoracic, transhiatal, and transcervical techniques—we highlight the implications of variations for intraoperative safety and postoperative outcomes. A thorough understanding of these relationships is essential for surgical planning, minimizing morbidity, and achieving oncological outcomes. Ultimately, a thorough understanding of these relationships is essential for patient-tailored surgical planning.

## 1. Introduction

Esophageal cancer is a major burden, ranking ninth in incidence and sixth in cancer-related mortality worldwide [1,2]. Management of esophageal cancer has undergone substantial evolution, shifting from primarily surgical resection to a multimodal framework that combines chemotherapy, radiotherapy, and refined surgical strategies [1].

Surgery remains the curative treatment, but esophagectomy is among the most complex procedures in gastrointestinal oncology. Historically, transhiatal and transthoracic esophagectomies represented competing approaches, each with distinct advantages and risks [3]. Randomized trials demonstrated that the transhiatal approach reduced perioperative morbidity, whereas an extended transthoracic resection with systematic mediastinal lymphadenectomy provided superior oncological clearance and a trend toward improved long-term survival [3]. Minimally invasive techniques have further transformed surgical practice. The literature shows that totally minimally invasive esophagectomy reduces pulmonary complications compared with open surgery [2]. Large single-center series and multicenter randomized trials have subsequently confirmed the safety and efficacy of minimally invasive and hybrid approaches, reporting reduced morbidity, particularly pulmonary complications, without compromising oncological outcomes [2,4]. Moreover, combined laparoscopic gastric mobilization with open thoracic resection offers a balance between reduced surgical trauma and technical reproducibility [4].

While personalized medicine in oncology has historically focused on individualized biomarkers and targeted systemic therapies, the paradigm has rapidly expanded to encompass precision surgery. In esophageal oncology, where the surgical field is exceptionally crowded, a structural approach is no longer sufficient. Because standardized anatomical textbook models frequently fail to account for unique patient geometry, modern esophagectomy must evolve into an individualized intervention. Transitioning to an era of patient-tailored surgery requires a profound appreciation of how structural anomalies dictate unique risk profiles for each patient.

As the surgical management of esophageal cancer continues to evolve, attention is increasingly focusing on anatomical precision. The esophagus traverses multiple body compartments and lies in intimate relation to critical vascular, neural, and lymphatic structures. Anatomical variations in these structures significantly influence surgical risk, the extent of resection, and postoperative morbidity. A detailed understanding of these variations and their implications for different surgical approaches is essential for optimizing outcomes. Therefore, we conducted a comprehensive review of the current literature up until November 2025 using the MEDLINE (PubMed) database. This narrative review examines the anatomical variability in the vessels, nerves, lymphatics, and their topographical relationships relevant to esophageal surgery, with a focus on the surgical risks and technical considerations.

## 2. Structures at Risk: Vessels and Their Variations

### 2.1. Variant Arterial Anatomy Related to the Esophagus

The esophagus is enveloped by a complex vascular network in the cervical, thoracic, and abdominal compartments. Many of these vessels follow highly variable courses, and their relationship to the esophageal wall and adjacent structures determines both their exposure and vulnerability during surgery.

**Figure 1:**
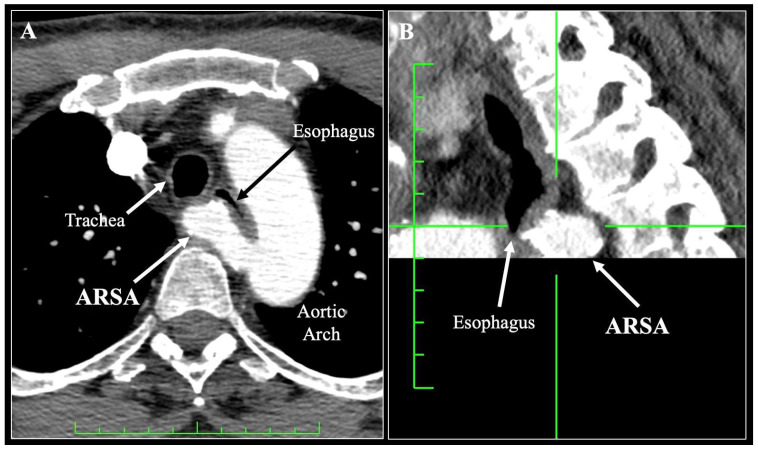
Computed tomography angiography of an aberrant right subclavian artery (ARSA) coursing posterior to the esophagus demonstrated in axial (**A**) and sagittal (**B**) reconstructions.

The most significant arterial variant is the aberrant right subclavian artery (ARSA), present in 0.16–4.4% of individuals [5,6]. Instead of arising from the brachiocephalic trunk, the ARSA originates as the last branch of the aortic arch, coursing posterior to the esophagus in 80–84% of cases, less commonly between the esophagus and trachea (12–15%), and rarely anterior to the trachea (≤5%) [5,6]. In its retroesophageal form (Figure 1) the ARSA crosses the midline at the level of the upper thoracic esophagus, lying between the esophageal adventitia and the vertebral column [7,8]. This intimate contact explains the characteristic posterior indentation of the esophagus seen on contrast studies [9]. Several case reports have described the preoperative identification of ARSA and its successful intraoperative management during esophageal cancer surgery [7,8,10,11,12].

**Figure 2:**
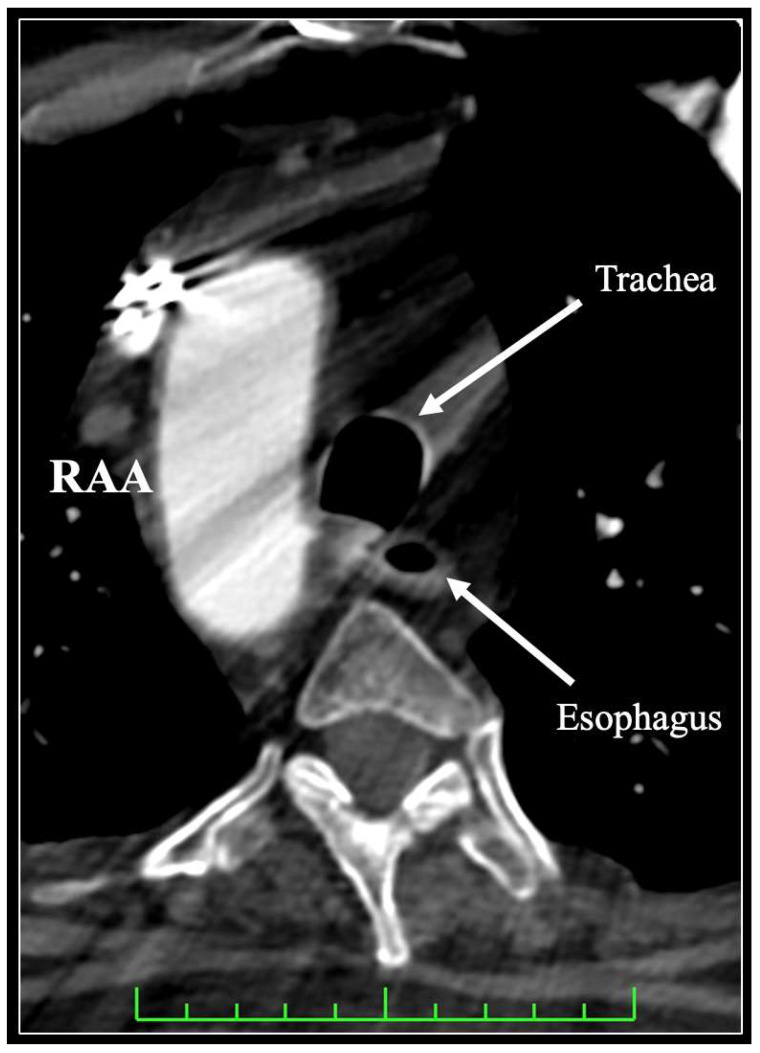
Computed tomography angiography of right-sided aortic arch (RAA) demonstrated in axial reconstruction.

Other aortic arch anomalies also modify the esophageal topography. A right-sided aortic arch may be associated with an aberrant left subclavian artery that follows a retroesophageal course (Figure 2), often arising from a Kommerell diverticulum [13].

The bronchial arteries (BAs) represent another frequent site of variation. They typically arise from the thoracic aorta at T5–T6 but may also originate from the intercostobronchial trunk, subclavian artery, or common trunks [14,15,16]. During an imaging study of esophageal cancer patients, right BAs arising from the intercostobronchial trunk were found to run along the right lateral aspect of the esophagus, dorsal to the trachea and bronchi. Left-sided BAs and common trunks more often coursed along the left margin of the esophagus [15]. This means that the esophagus serves as a central landmark, with BAs frequently crossing its adventitial plane, particularly in the middle mediastinum. Meta-analyses have revealed extreme variability in BA numbers (1–6 per patient) and laterality, with up to 41% arising separately from the aorta [14]. Because BAs also contribute branches to the esophagus itself and to periesophageal lymph nodes, their course is intimately tied to surgical planes in both subcarinal and paratracheal dissections [14].

### 2.2. Variant Venous Anatomy Related to the Esophagus

Venous drainage of the esophagus is equally intricate, reflecting its transitional location between the cervical, thoracic, and abdominal compartments. It communicates with systemic and portal venous systems, making its venous plexus clinically and anatomically significant.

**Figure 3:**
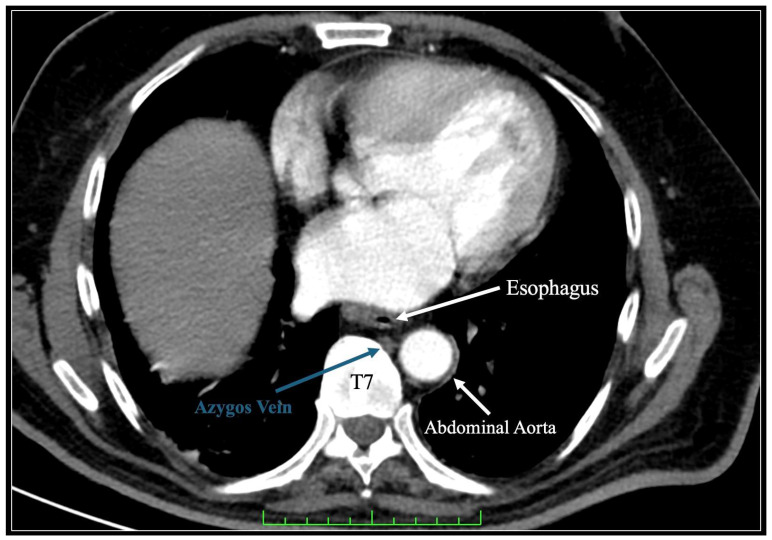
Computed tomography angiography of azygos vein, abdominal aorta and esophagus demonstrated in axial reconstruction.

The azygos vein (AV) is the dominant venous structure in relation to the thoracic esophagus. Running along the right side of the vertebral column, it ascends posterior to the esophagus and anteriorly over the right main bronchus to drain into the superior vena cava [17] (Figure 3). Numerous tributaries link it directly to the esophageal venous plexus, creating longitudinal anastomoses. On the left, the hemiazygos vein (HAV) and accessory hemiazygos vein ascend adjacent to the thoracic esophagus, frequently crossing its posterior surface to join the AV through retroaortic anastomoses [18]. These crossings occur at varying thoracic levels (commonly T7–T9), lying in close contact with the esophageal adventitia. Meta-analyses have confirmed high variability in these anastomoses, with type II (multiple retroaortic connections between the AV and HAV) being the most prevalent pattern [17]. Rarely, the AV may serve as a continuation of the inferior vena cava, coursing posterior to the esophagus as its primary drainage route [19]. In such cases, the vein is often enlarged, tightly adherent to the posterior esophageal wall, and cannot be sacrificed without major hemodynamic consequence.

## 3. Structures at Risk: Nerves and Their Variations

At the level of the distal trachea and proximal esophagus, the right and left vagus nerves contribute numerous branches, forming the esophageal plexus. These fibers, interwoven on the adventitia, ultimately reconstitute into anterior and posterior vagal trunks, which continue along the abdominal esophagus. This plexus represents a constant feature, though its density and lateral spread vary, sometimes enveloping more of the lateral esophageal wall than the posterior aspect [20].

### 3.1. Recurrent Laryngeal Nerves

The right vagus nerve gives off the recurrent laryngeal nerve (RLN) near the brachiocephalic–subclavian junction. The RLN hooks around the subclavian artery (or distal brachiocephalic trunk) and ascends cranially. It re-enters the neck through the tracheoesophageal groove, running close to the cervical esophagus before penetrating the larynx [20]. The left RLN originates from the vagus at the aortic arch, looping under it just posterior to the ligamentum arteriosum, then coursing back cranially in the left tracheoesophageal groove, in close proximity to the cervical and upper thoracic esophagus [21].

Because of their looping trajectories, the RLNs cross the mediastinum and ascend immediately adjacent to the esophagus and trachea. The left RLN, with its longer intrathoracic course, maintains more intimate contact with the esophageal wall than the right [22].

A non-recurrent RLN is a rare variant, typically presented on the right and associated with an ARSA. In this case, the nerve arises directly from the vagus and courses transversely, without descending into the mediastinum [20].

Both RLNs give off multiple twigs (8–14 branches) to the trachea and esophagus. These branches frequently interconnect, forming a peritracheoesophageal neural network [22].

Cadaveric studies have shown that the left RLN may branch from the vagus above the arch (11%), at arch level (48%), or below it (41%), altering its relation to the esophagus and ligamentum arteriosum [21].

RLNs are most often found within the tracheoesophageal groove (approximately 64%), but can also lie lateral, anterolateral, or anterior to it. The left nerve tends to be deeper and more medial, but overall, the right and left RLN locations do not differ significantly at cervical levels [23,24]. This variability makes their exact position in relation to the esophagus unpredictable.

### 3.2. Superior Laryngeal Nerves

The superior laryngeal nerves (SLNs), branching directly from the vagus at higher cervical levels, descend toward the larynx and pharynx. Their external branches run obliquely across the upper cervical esophagus, in close proximity to the pharyngoesophageal junction, where they may overlap with fibers from the RLNs [22]. The dual innervation from RLN and SLN fibers provides functional redundancy but also increases the density of neural twigs surrounding the proximal esophagus [22].

### 3.3. Sympathetic Chain and Periesophageal Nerves

The thoracic sympathetic trunk runs laterally to the vertebral bodies, but thin visceral branches course medially toward the esophagus, particularly at midthoracic levels. These fibers contribute to the esophageal autonomic plexus in combination with the vagal branches. Their course is less consistent, but they frequently cross the posterolateral aspect of the thoracic esophagus within the loose connective tissue [25].

## 4. Structures at Risk: Lymphatics and Their Variations

The lymphatic system associated with the esophagus is dominated by the thoracic duct (TD) and its tributaries, which run in a close topographical relationship with the esophagus throughout the posterior mediastinum. Its variations, duplications, and plexiform formations account for much of the anatomical complexity encountered during esophageal surgery.

The TD is formed embryologically from paired right and left lymphatic trunks with multiple anastomoses. Selective regression usually leaves the inferior part of the right trunk and superior part of the left trunk, linked by a transverse anastomosis at T4–T6 [26,27]. This developmental origin explains the frequent persistence of duplications or plexiform networks seen in adults [28].

Classically, the duct arises from the cisterna chyli (L1–L2), ascends through the aortic hiatus of the diaphragm, and courses in the posterior mediastinum between the descending aorta (left) and AV (right), dorsal to the esophagus [29]. Around T5, it crosses obliquely to the left side, continuing cephalad along the left lateral aspect of the esophagus before curving anteriorly in the root of the neck to enter the left jugulo-subclavian venous angle [27].

Plexiform or duplicated ducts are present in 10–20% of individuals; these ducts consist of a plexus or partial duplication, sometimes persisting as V-shaped or inverted V-shaped configurations [28].

Multiple tributaries above the diaphragm have been depicted through cadaveric studies; these have shown, in most cases, that the duct is formed by 3–5 abdominal tributaries that converge just above the aortic hiatus rather than in the abdomen [30,31].

Complete duplication is rare (<2%) but is a possible anatomical variation, with right and left channels persisting throughout their course and draining separately into the venous system [31].

In the posterior mediastinum, the duct usually ascends to the right of the esophagus until T5, where it crosses posterior to the esophagus to the left side [29]. In some individuals it remains on the right side for its entire course, draining into the right venous system [32]. Networks of small lymphatic channels may run directly on the esophageal wall, particularly near the aortic arch, over the adventitia [32].

A combined histological and imaging study demonstrated that the periesophageal connective tissue contains lymphatic vessels and nodes embedded in fascial planes [33]. A distinct aortoesophageal ligament was described, which links the descending aorta to the esophagus and contains small vessels, lymphatics, and sometimes nodes [33]. This structure separates the anterior periesophageal compartment (esophagus, vagus, and tracheobronchial nodes) from the posterior para-aortic compartment (AV, TD, and posterior mediastinal nodes) [33].

In the lower cervical region, the TD courses 3–4 cm above the clavicle, coursing posterior to the carotid sheath and anterior to the subclavian artery before entering the venous system [29]. Small cervical lymph nodes (e.g., Virchow’s node at the left jugulo-subclavian junction) form the key landmarks in this area [26].

## 5. Structures at Risk: Fasciae, Ligaments and Muscles

### 5.1. Fasciae and Ligaments—Observations from Surgical Studies

The esophagus is embedded in distinct fascial structures that separate it from adjacent mediastinal compartments. Cuesta et al. [34] introduced the concept of the mesoesophagus, a fascia-like envelope extending from the thoracic inlet to the diaphragmatic hiatus, which is especially prominent between the esophagus and descending aorta [34]. This fascia contains esophageal vessels, lymphatics, and nerves and provides a connective tissue plane analogous to the mesorectum. Embryological studies have confirmed that the esophagus is initially suspended in a mesentery-like structure, which persists into postnatal life as a vestigial fascial plane along the lower thoracic esophagus [35]. More recently, Cuesta et al. [36] introduced a similar concept of the mesoesophagus at the supracarinal level.

Complementary cadaveric and thoracoscopic studies have further defined two main connective tissue layers: (i) the aortoesophageal ligament, tethering the esophagus to the descending aorta, and (ii) the aorto-pleural ligament, linking the aorta to the pleura [33,37]. These ligaments create distinct periesophageal (esophagus, vagus nerves, and tracheobronchial nodes) and para-aortic (AV and TD) compartments [33,38].

**Figure 4:**
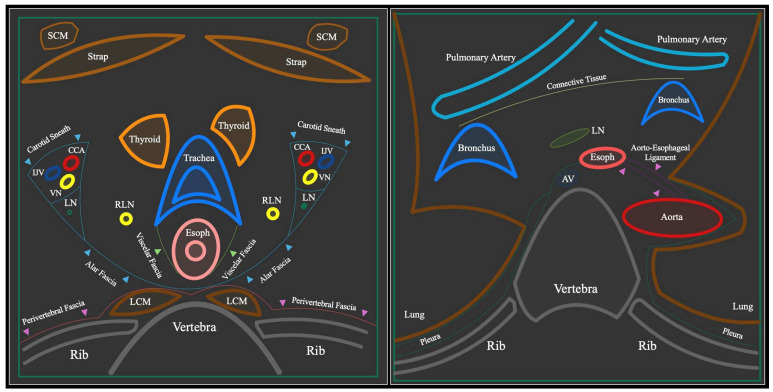
Schematic representation of the topographical relationship at the thoracic inlet (**left**) and the upper mediastinum (**right**) (adapted from Weijs et al. (2017)) [33]. Esoph—esophagus; CCA—common carotid artery; IJV—internal jugular vein; VN—vagus nerve; LN—lymph node; RLN—recurrent laryngeal nerve; SCM—sternocleidomastoid muscle; LCM—longus colli muscle.

At the thoracic inlet, the esophagus lies posterior to the trachea and medial to the carotid sheaths. The carotid sheath, intercarotid fascia, and visceral fascia provide useful dissection landmarks, with the RLNs, TD, and sympathetic trunks embedded in close proximity [33,39]. The esophagus descends just left of the midline, bounded dorsally by the vertebral column and prevertebral fascia (Figure 4).

In the upper mediastinum, the esophagus is flanked anteriorly by the trachea and left main bronchus; laterally by the pleura and great vessels—the aortic arch and pulmonary arteries on the left, and the azygos system on the right; and posteriorly by the TD and descending aorta [33] (Figure 4). The AV typically goes over the right main bronchus to join the superior vena cava, crossing dorsal to the esophagus. Variants, such as an azygos lobe—a congenital anomaly where the AV courses abnormally within the lung apex—alter this relationship, potentially narrowing the operative corridor [40].

3D reconstructions of transcervical approaches have highlighted four constant landmarks for orientation: (i) the TD course, (ii) left RLN, (iii) AV arch dorsal to the esophagus, and (iv) aortic arch and pulmonary vessels [38]. Below the carina, the esophagus runs posterior to the left atrium, with the pericardium and pulmonary veins anteriorly, and the descending aorta to the left. The mesoesophagus in this region harbors subcarinal lymph nodes, bronchial branches, and periesophageal vascular connections [34].

The cervical esophagus transitions into the thoracic cavity at the thoracic inlet, where the narrow operative field exposes multiple structures at risk. On the left side, the esophagus is tethered to the aortic arch via the aortoesophageal ligament, while on the right side it is bordered by the AV and vagus nerve [39].

### 5.2. Fasciae and Ligaments—Observations from Histological Studies

In parallel with these important studies, the Tokairin et al. [41,42,43,44] research group has significantly expanded our understanding of the periesophageal landscape. Their histological and embryological work has shifted the paradigm from cylindrical sheaths to a more complex, asymmetrical microscopic anatomy dictated by mechanical forces like pulsation and peristalsis.

The upper mediastinum is characterized by histologically identifiable visceral and vascular sheaths that do not maintain the perfectly cylindrical geometry described in earlier gross anatomical models [44]. These thin membranous dense connective tissues exhibit a distinct left–right asymmetry, where the visceral sheath is more prominently developed on the left side of the esophagus while remaining notably indistinct on the right [44]. Its clinical significance is particularly high near the tracheal bifurcation, where the visceral sheath gradually thins and almost disappears. In this specific zone, the esophagus attaches to the TD with such minimal intervening tissue that the optimal dissection layer becomes difficult to define, increasing the risk of unrecognized injury [44].

In the middle and lower mediastinum, the architecture of these layers shifts as the visceral sheath transitions into a mesentery-like structure [43]. This configuration consists of integrated dorsal and ventral layers of dense connective tissues that extend bilaterally to anchor the esophagus to the pulmonary hila and the left sub-pleural region [43]. The posterior component of this visceral sheath is histologically identified as the inter-pleural ligament (Morosow’s ligament), which provides a key surgical plane for separating the esophageal adventitia from the descending aorta, AV, and TD [43]. Within this region, the vascular sheath remains a separate entity, primarily connecting the great vessels and originating from the connective tissue in front of the vertebral bodies [43].

The relationship between these fascial layers and the RLNs is most dynamic at the curving portions of the nerves near the aortic arch and right subclavian artery [42]. A histological examination revealed that the visceral sheath is not clearly observed at the actual site of inversion, meaning there is no distinct membranous boundary between the esophagus and the lymph node stations in this region, such as the left tracheobronchial or right recurrent nerve nodes [42]. Consequently, these nodes reside in the same compartment as the esophagus and trachea at the curving point, whereas stations like the right cervical paraesophageal nodes are clearly medial to the visceral sheath [42].

Finally, the most recent embryological analysis suggested that these sheaths are not pre-formed templates but rather products of developmental maturation influenced by mechanical forces [41]. While only homogeneous loose fibrous tissue is present at four months of gestation, distinct density differences in collagen fibers begin to emerge by eight months, forming the structural basis for adult visceral and vascular sheaths [41]. The development of the vascular sheath appears to be driven by the pulsations of the great thoracic vessels, which explain its earlier and more robust appearance [41]. Conversely, the full maturation of the visceral sheath is likely dependent on the mechanical stimuli of esophageal peristalsis and swallowing movements that occur postnatally, explaining why these layers are less distinct in fetal specimens [41].

### 5.3. Muscles and Ligaments

Within the posterior mediastinum, the pleuroesophageal muscle represents a frequently disregarded yet surgically relevant smooth muscle slip [45]. Extending from the left mediastinal pleura and typically crossing anterior to the thoracic aorta, the pleuroesophageal muscle attaches to the longitudinal fibers of the esophagus, often at the level of the seventh thoracic vertebra [45]. This muscle, along with similar slips and the phrenicoesophageal ligament, acts as an anchoring structure that provides fixed points for the esophagus [45,46]. These points facilitate effective contraction during swallowing and ensure the esophagus returns to its baseline position following the descent of the diaphragm during inspiration [45]. For surgeons, these structures represent a substantial risk during mobilization. A prominent muscle can reach lengths of 37.6 mm and widths of 10 mm [45]. Forceful or rapid traction on these dense anchoring bands during blunt or robotic dissection can result in significant tissue tearing at the points of attachment, potentially compromising the integrity of the esophageal wall or the mediastinal pleura [45].

## 6. Surgical Considerations for Different Approaches

As previously demonstrated, the esophagus lies within one of the most anatomically crowded regions of the body, and the choice of surgical approach—right transthoracic, left transthoracic, transhiatal, or cervical—dictates which anatomical variations come most prominently into the surgeon’s view. Each route to the esophagus exposes the surgeon to a distinct set of vascular, neural, and lymphatic hazards. Understanding how these structures may deviate from their expected course is essential to anticipate risks and adapt intraoperative strategies.

### 6.1. Right Transthoracic Approach

**Figure 5:**
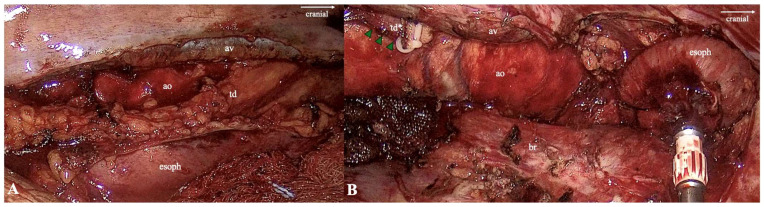
Intraoperative images from a right transthoracoscopic approach illustrating the anatomical structures at risk—the thoracic aorta (ao), the azygos vein (av) and the thoracic duct (td)—along with the esophagus and the bronchus (br). In the left image (**A**) the av and td are dissected, while in the right image (**B**) the td is ligated (*) and the av is divided.

In a right thoracoscopic or robotic esophagectomy, the surgeon works predominantly within the posterior mediastinum. Here, the esophagus is suspended between two connective tissue layers: the aortoesophageal and aorto-pleural ligaments. These natural fascial boundaries divide the mediastinum into a periesophageal compartment—containing the vagus nerves, carinal lymph nodes, and trachea—and a para-aortic compartment harboring the TD and AV [37] (Figure 5).

Adhering to the periesophageal plane reduces the risk of inadvertent entry into the para-aortic space, where fragile lymphatic and venous structures predominate [34,36]. The TD is the principal structure at risk in this field. Although classically it crosses from right to left behind the esophagus at the level of T5, its course is highly variable. In up to 14% of patients it may remain entirely on the right, or it may exist as a plexiform or duplicated system [28,30]. At the level of T9–T10, the duct usually lies between the right border of the aorta and the left anterolateral aspect of the AV, providing a consistent landmark for identification and safe ligation [29]. However, just above the hiatus, multiple tributaries frequently converge rather than forming a single trunk, meaning that a simple ligation at the hiatus can leave parallel channels intact [27,30]. Indocyanine Green (ICG) can be used during a trans-abdominal approach (with its injection into the mesentery of the small bowel), and the TD can be easily identified during a transthoracic (or transthoracoscopic) approach [47].

The azygos system itself introduces variability. While the AV arch normally crosses over the right main bronchus to join the superior vena cava, it may form high or low arches, duplications, or even serve as a continuation of the inferior vena cava. In such cases, the vessel runs immediately behind the esophagus as its principal venous drainage route [19]. An injury in this setting is catastrophic. The BAs also complicate the thoracic phase. These vessels frequently cross the lateral esophageal adventitia and show variable origins, including from the intercostobronchial trunk or directly from the thoracic aorta [15]. Sacrificing all of them during subcarinal dissection may compromise bronchial and esophageal perfusion [48].

Finally, anomalies of the great vessels at the thoracic inlet present hazards during a right transthoracic approach. An ARSA, most commonly coursing posterior to the esophagus, can compress the posterior wall and alter the dissection planes [5].

### 6.2. Left Transthoracic Approach

In a left-sided esophagectomy, the esophagus is approached through the aortopulmonary window and upper mediastinum. The most critical structure here is the left RLN. Its point of origin from the vagus is variable: in some patients it separates above the aortic arch, in others at the arch itself, and in the remainder just below it [21]. It invariably passes posterior to the ligamentum arteriosum, which therefore serves as a reliable landmark. The nerve ascends in the left tracheoesophageal groove, but the width of this groove and the position of the nerve within it vary, necessitating its cautious identification before proceeding with a lymphadenectomy in stations 4L, 5, and 6 [23,24].

Additional risks arise from vascular variation. Large BAs or accessory left bronchial trunks may overlie the esophagus in the window, and their preservation can be difficult when clearing peribronchial tissue [14]. The TD is also inconsistent at this level. Rather than crossing at T5 in a predictable fashion, it may lie medial, anterior, or posterior to the left subclavian artery, and in some cases has no direct relationship with the arch at all [29]. This variability broadens the zone of risk during upper mediastinal dissections.

### 6.3. Transhiatal Approach

The transhiatal route avoids a thoracotomy but requires blunt dissection through the posterior mediastinum from both the cervical and abdominal directions. The risk here lies in the absence of direct visualization. The aortoesophageal ligament marks the posterior boundary of the periesophageal compartment, and violation of this plane brings the surgeon into the para-aortic space, where the TD and AV run [34].

At the diaphragmatic hiatus, the TD is particularly variable. Rather than entering through the hiatus as a single cisterna chyli, multiple lymphatic tributaries often converge just above the diaphragm [30]. Surgeons who attempt to ligate the duct at the hiatus may therefore miss parallel channels. In rare cases, azygos continuation of the inferior vena cava also presents as an enlarged vessel immediately posterior to the esophagus, complicating blunt cephalad dissection [19]. These anomalies explain the high risk of unrecognized lymphatic or venous injury in transhiatal procedures.

### 6.4. Cervical and Transcervical Approaches

Cervical mobilization, as in the McKeown procedure or a transcervical esophagectomy, places the RLNs and terminal TD at greatest risk. The RLNs normally ascend in the tracheoesophageal grooves, but both nerves may exhibit extralaryngeal branching, meandering courses, or variable relationships with the inferior thyroid artery [22,23]. A non-RLN, most often right-sided and associated with an ARSA, lies transversely across the root of the neck rather than descending into the mediastinum, creating a high risk of inadvertent division [20].

Transcervical mediastinal approaches, such as robot-assisted or mediastinoscopic dissections, have mapped reproducible landmarks down to the carina: the left RLN, TD, azygos arch, and aortic arch together define the safe corridor [38]. Even so, the termination of the TD remains unpredictable. In the vast majority of cases it drains into the left jugulo-subclavian venous angle, but in up to 80% of cases there are two or more terminal channels, and in rare instances the duct empties into the right side or bilaterally [31,49]. Dissection in the root of the neck must therefore be layered and cautious to avoid an unrecognized lymphatic injury.

### 6.5. Postoperative Complications

Anatomical variations in the mediastinal corridor significantly elevate the risk of intraoperative hemorrhage and postoperative vascular complications [50]. The presence of an ARSA typically courses posterior to the esophagus, creating an unpredictable arterial landmark that can be injured during esophageal mobilization.

The risk of chylothorax is largely driven by the plexiform nature of the TD and its shifting relationship with the fascial sheaths. Because the TD exists as a duplicated or plexiform system, the ligation of a single visible trunk may leave parallel lymphatic channels intact, resulting in a persistent postoperative leak [51].

An RLN injury, which can lead to permanent vocal cord paralysis and aspiration, is a frequent complication due to the highly variable neural pathways [52]. A non-recurrent RLN (associated with an ARSA) does not descend into the mediastinum but instead crosses transversely in the neck, making it extremely vulnerable to inadvertent division during cervical mobilization [52].

### 6.6. Preoperative Preparation

Across all approaches, preoperative imaging has become indispensable. High-resolution CT angiography delineates aberrant arteries and veins, while MR thoracic ductography can identify the side of ductal ascent and major duplications in over 90% of patients [32]. Baiwei et al. [53] recorded aortic arch variants during surgical treatment of esophageal cancer. They highlighted that preoperative evaluation and adequate surgical approach selection should be carefully considered [53].

Endoscopic ultrasonography (EUS) is another vital tool for the preoperative staging of esophageal cancer to determine whether locally advanced tumors require neoadjuvant therapy prior to surgical intervention [54,55]. By providing high-resolution images, EUS facilitates a deeper understanding of the esophageal wall layers and surrounding landscape, which is essential for safe dissection and achieving curative oncological outcomes [54,55].

Nowadays, three-dimensional reconstructions of a patient’s scans along with artificial intelligence can be used to create a unique anatomical model of the patient’s anatomy preoperatively [56]. Hence, integrating advanced technologies during surgery will help to provide adequate knowledge of the personalized anatomy of each patient.

The application of a multidisciplinary team is central to the modern multimodal framework for treating esophageal cancer, which has evolved from a primarily surgical focus to an integrated strategy combining chemotherapy, radiotherapy, and surgery [57,58]. During preoperative preparation, the team evaluates the tumor histology and stage to determine if locally advanced tumors require neoadjuvant therapy before resection [57]. A critical component of this collaborative planning involves meticulous imaging and technological integration [57]. In this context, the team utilizes high-resolution CT, EUS and MR lymphangiography to identify significant anatomical variations.

## 7. Conclusions

Esophageal surgery is uniquely defined by its anatomical complexity. The esophagus traverses the neck, chest, and abdomen, surrounded by major vessels, nerves, and variable lymphatic pathways. Variations in these structures are common rather than exceptional, and their presence alters the operative planes, increases the risk of injury, and influences both the safety and completeness of resection. The recognition of these patterns is transitioning esophageal oncology away from rigid, conventional surgical dogmas into an era of personalized surgical medicine. These patterns require a dual strategy, with meticulous preoperative imaging, including high-resolution CT and MR lymphangiography, to anticipate anomalies. Intraoperative adherence is also significant for compartment-based surgical planes with careful identification of at-risk structures. Across all surgical approaches—whether transthoracic, transhiatal, or transcervical—the outcomes depend on tailoring the technique to an individual’s anatomy. Ultimately, mastering the spectrum of anatomical variations and translating them into patient-specific roadmaps will be integral to achieving safe, radical, and function-preserving personalized esophageal cancer surgery.

## Data Availability

No new data were created or analyzed in this study. Data sharing is not applicable to this article.

## Abbreviations

The following abbreviations are used in this manuscript:

ARSA	Aberrant right subclavian artery
BA	Bronchial artery
AV	Azygos vein
RLN	Recurrent laryngeal nerve
SLN	Superior laryngeal nerve
TD	Thoracic duct
